# Double-walled iron oxide nanotubes via selective chemical etching and Kirkendall process

**DOI:** 10.1038/s41598-019-47704-5

**Published:** 2019-08-19

**Authors:** João Azevedo, M. P. Fernández-García, César Magén, Adélio Mendes, João P. Araújo, Célia T. Sousa

**Affiliations:** 1 LEPABE - Faculdade de Engenharia, Universidade do Porto, Rua Dr. Roberto Frias, 4200-465 Porto, Portugal; 2 IFIMUP and Departamento de Física e Astronomia da Faculdade de Ciências da Universidade do Porto, Rua do Campo Alegre 687, 4169-007 Porto, Portugal; 3 Instituto de Ciencia de Materiales de Aragón (ICMA), Universidad de Zaragoza-CSIC, 50009 Zaragoza, Spain; 4 Laboratorio de Microscopías Avanzadas (LMA) - Instituto de Nanociencia de Aragón (INA), Universidad de Zaragoza, 50018 Zaragoza, Spain; 5 Departamento de Física de la Materia Condensada, Universidad de Zaragoza, 50009 Zaragoza, Spain; 6 Department of Physics, University of Oviedo, 33007 Oviedo, Spain

**Keywords:** Nanoscale materials, Materials for energy and catalysis

## Abstract

Double-walled oxide nanotube structures are interesting for a wide range of applications, from photocatalysis to drug delivery. In this work, a progressive oxidation method to fabricate double-walled nanotube structures is reported in detail. The approach is based on the electrodeposition of metallic iron nanowires, in porous alumina templates, followed by a selective chemical etching, nanoscale Kirkendall effect, a fast oxidation and out-diffusion of the metallic core structure during thermal annealing. To validate the formation mechanism of such core-shell structure, chemical composition and atomic structure were assessed. The resulting hematite nanotubes have a high degree of uniformity, along several microns, and a nanoscopic double-walled structure.

## Introduction

Iron oxides have attracted much interest for their potential application in nanotechnology^1–4^. In particular hematite (α-Fe_2_O_3_), being the most stable form among several Fe oxide phases^5^, is of significant scientific and technological importance for its wide application in catalysis/photocatalysis^6,7^, gas sensors^8^ and drug delivery^9^. One of the greatest challenges with this material is its nanostructuring, which is required to increase the surface area for better reaction kinetics^5,10^, light absorptivity^6,11^ or electronic conductivity^12^. Thus, low-cost and scalable nanostructuring production methods are essential challenges in which researchers are focusing on, so that the full potential of this material is exploited.

Various α-Fe_2_O_3_ nanostructures with different morphologies such as nanoparticles^8,12^, nanowires (NWs)^13,14^ and nanotubes (NTs)^15,16^ have already been successfully synthesized. Additionally, several works reported, recently, the synthesis of hollow iron oxide nanoparticles through the Kirkendall effect^13–17^. The major part of these works refers to the fabrication of hollow spherical magnetite nanoparticles, but also nanorods and nanocubes were reported. These hollow structures are an opportunity for constructing complex nanodevices, but the reported methods were limited to spherical (or quasi-spherical) nanoparticles with many difficulties in the precise control of the final dimension and morphology of the nanostructure. Given that fundamental properties of materials can be tuned through morphology and size, precise structure control is of utmost importance^18,19^. For example, nanostructured α-Fe_2_O_3_ has been studied as an anode material for lithium ion batteries for its high capacitance and it was demonstrated that higher porosity is advantageous over repetitive cycling^15^; this way a nanotube would provide better results than a nanorod, for example. Also, quantum confinement in very thin nanostructures, such as nanotubes with extremely thin walls, can be used to tune the band edges and band gap of a semiconductor for photoelectrochemical applications^19^. In this way, template-assisted methods are advantageously providing a highly flexible and controlled template that shapes the desired nanoparticle to the intended dimensions. Some reports have been published on the synthesis of α-Fe_2_O_3_ NTs using template-assisted methods^20,21^, but no report of a double-walled NT structure using such methods has been made. Moreover, the reported nanostructures were relatively wide in diameter (>100 nm), decreasing the attainable surface-to-volume ratio. The reason for this is the difficulty to make a controlled chemical etching to erode the nucleus and leave the walls intact.

A novel and scalable fabrication method of crystalline α-Fe_2_O_3_ double-walled NTs grown on porous anodic alumina (PAA) templates with controllable dimensions is here reported. For the first time, 1D Fe nanostructures grown inside PAA templates were converted into the desired α-Fe_2_O_3_ phase with double walled NT morphology by combining a controlled selective chemical etching and the Kirkendall effect through an annealing route. A detailed morphological analysis was performed to reveal the growth mechanism of double-walled hollow nanostructures. The key role of the Kirkendall effect, to obtain double-walled nanostructures instead of reported single-walled, is here addressed.

## Results and Discussion

### Double walled hematite nanotubes

**Figure 1:**
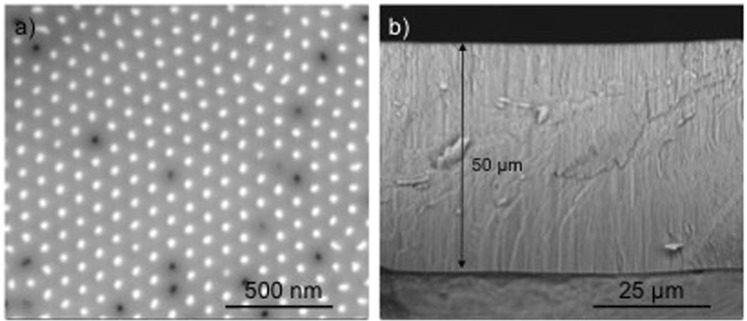
(**a**) Top view SEM images showing the high-density of Fe nanowire array. (**b**) Cross section reveals the thickness of the template, without any cracks.

Crystalline Fe NWs were grown inside PAA templates into pores of 35 nm diameter, typical from oxalic acid self-organized regime anodization (Fig. 1a). Oxalic acid was used due to its high reproducibility rate and because it yields nanometric pore diameter necessary for high surface areas^22^. Other pore sizes are currently under study and not reported in this work. The deposition was highly homogeneous up to lengths of 50 μm, which were used to achieve large aspect ratios (>1.4 × 10^3^). Cross-section scanning electron microscopy (SEM) images revealed no defects along the PAA template and each wire was continuous and homogeneous (Fig. 1b).

The structure and phase composition of the samples were determined by X-ray diffraction (XRD) (Fig. S2). The positions of all Bragg reflections in Fe NWs coincide with the Bragg reflections characteristic of metallic α-Fe phase. Metallic Fe NWs show a preferential bcc-structure and only two peaks, (110) and (200), can be found in the patterns. From the most intense peak (110), a grain size of 17.8 nm was estimated.

The encapsulated NWs were removed from the PAA template, using a chemical etching solution containing H_3_PO_4_, known to promote selective coordination-assisted dissolution, which is discussed in detail later on in this paper. The Bragg reflections of the Fe NWs after template removal were broader than in template (Fig. S2), since the vertical alignment provided by the PAA was lost with its dissolution and the NWs became dispersed. The relative size of the peaks also changed, related to textured NWs in template versus more polycrystalline configuration in dissolved NWs.

**Figure 2:**
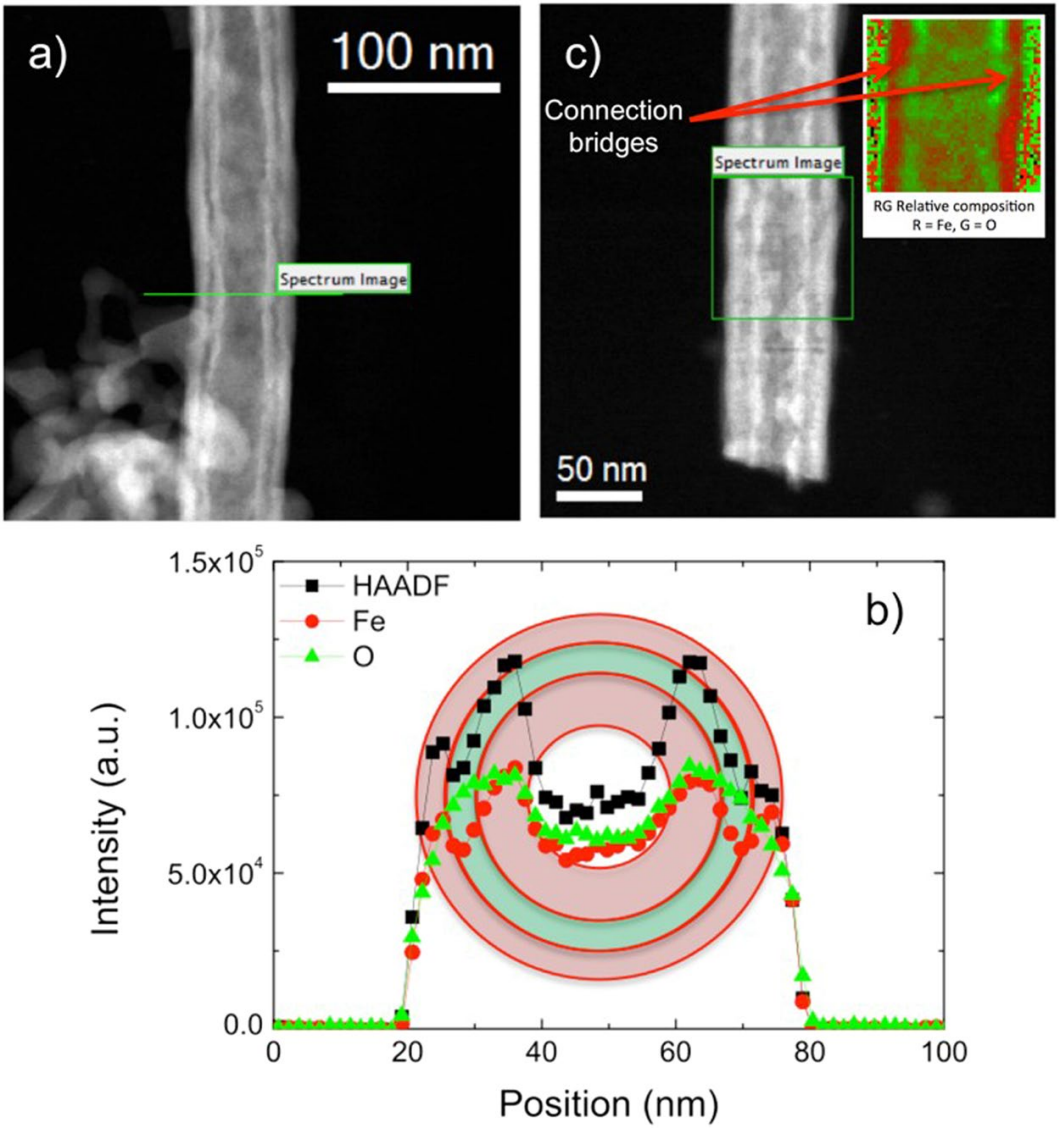
(**a**) High angle annular dark field (HAADF) STEM side-view image of a single double walled nanotube; (**b**) STEM-EELS chemical profile across α-Fe_2_O_3_ double wall NT along the green line in (**a**); (**c**) HAADF-STEM side-view of another double walled nanotube with composition on inset.

The NWs were annealed at 600 °C in air during 6 h. The morphology of the obtained nanostructures was analyzed by scanning transmission electron microscopy (STEM) and a double-walled nanotube structure was observed – Fig. 2a. The electron energy loss spectroscopy (EELS) compositional line profiles of this double-walled NTs indicate Fe and O signals variation consistent with the double-walled structure – Fig. 2b. From the compositional line profiles, a distance between inner and outer tubes of ca. 4 nm, with corresponding diameters of 21 nm and 46 nm, was estimated. Inner and outer walls have different thicknesses (8 nm and 4 nm, respectively), which is 5 times thinner than previous reports, explained by the small features of the selected PAA template, providing a much larger surface-to-volume ratio^13^. Very thin walls are interesting for different applications due to the quantum confinement effects^19^. The NTs bottom ends (designated as the side originally adjacent to the Al foil before removing the PAA template) were dissolved during the alumina chemical etching, so no dendritic structure was present and most NTs were opened/broken at both ends. The inner and outer tubes do not appear to be connected at any specific point and only very thin connection bridges were observed – Fig. 2c. These double-walled nanotubes present a higher geometrical surface area than other reported nanostructures – Fig. S1.

**Figure 3:**
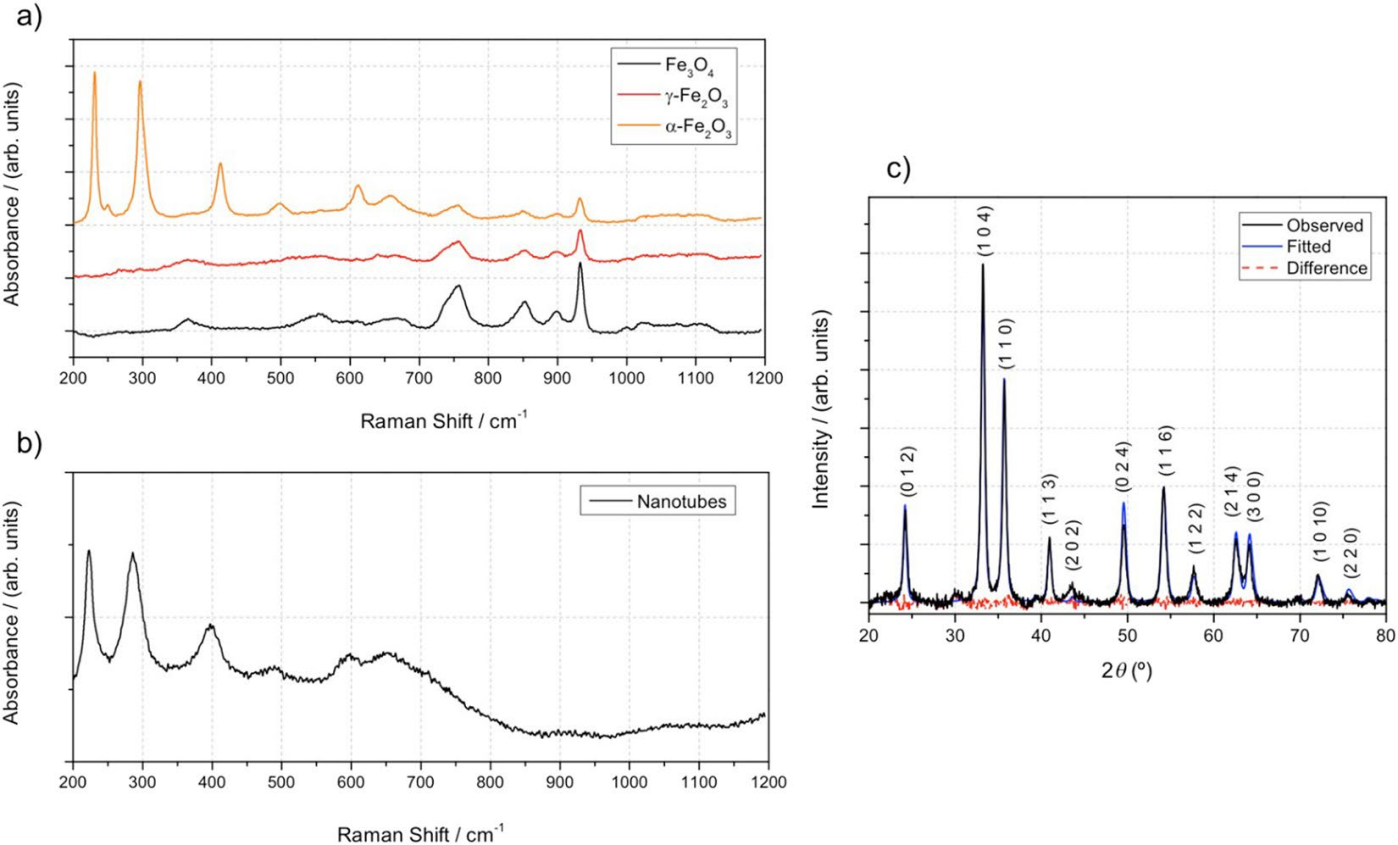
(**a**) Raman spectra of three most common iron oxide phases; (**b**) Raman spectra of double walled nanotubes (peaks fit well with hematite); (**c**) X-ray diffraction patterns of double walled nanotubes, with the corresponding miller indexes are marked. Peak values were compared to reference – Table S1.

The composition and crystallographic structure of the NTs were assessed by different techniques. Raman spectroscopy was performed on reference samples of the most common Fe oxides (maghemite, magnetite and hematite) - Fig. 3a. The NT modes positioned at 221, 285, 397, 602 and 645 nm in the spectrum (Fig. 3b) are all indexed to the characteristic bands of α-Fe_2_O_3_ confirming that the NTs are constituted of only one Fe oxide phase – α-Fe_2_O_3_. The XRD pattern of the NTs is shown in Fig. 3c. All the Bragg reflections were identified and correspond to the α-Fe_2_O_3_ phase. The spectrum was fitted with hematite (PDF Card No.: 2101167) and is in great accordance with the α-Fe_2_O_3_ spectrum. All XRD peaks can be indexed to α-Fe_2_O_3_ R-3C hexagonal cell with lattice constants of a = b = 5.0380 Å and c = 13.7662 Å. A grain size of 11.5 nm was determined using the Scherrer equation.

**Figure 4:**
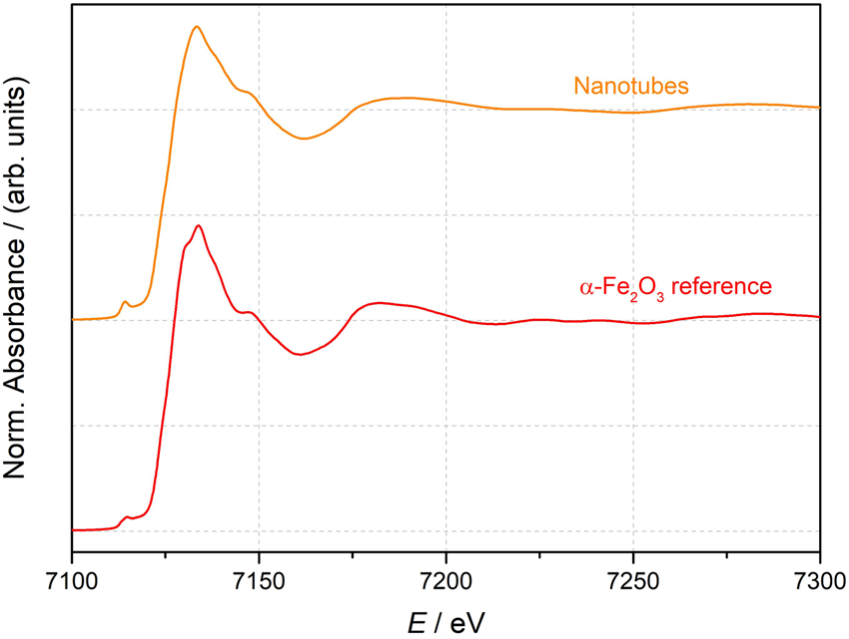
Experimental Fe K-edge XANES spectrum of a double walled nanotube sample (orange line) measured at room temperature together with the hematite reference (red line).

To confirm the composition of the nanostructures in a precise manner, X-ray absorption spectroscopy (XAS), a technique selective to atomic species, was employed. The existence of Fe hydroxides (goethite or ferrihydrite) magnetite and maghemite was discarded by both XRD and Raman spectrometry and therefore, only hematite was considered for the data analysis of the X-ray absorption spectroscopy near edge structure (XANES) spectrum. In Fig. 4 the experimental Fe K-edge XANES spectrum of the sample is shown together with the reference of α-Fe_2_O_3_ measured in the same equipment. It can be observed that the experimental spectrum reproduces the shape, energy position of the different spectral features, their relative energy separation and the intensity ratio of the reference sample. This result is in accordance with the XRD measurements, validating the oxidation of the nanostructure to α-Fe_2_O_3_.

### Growth Mechanism

**Figure 5:**
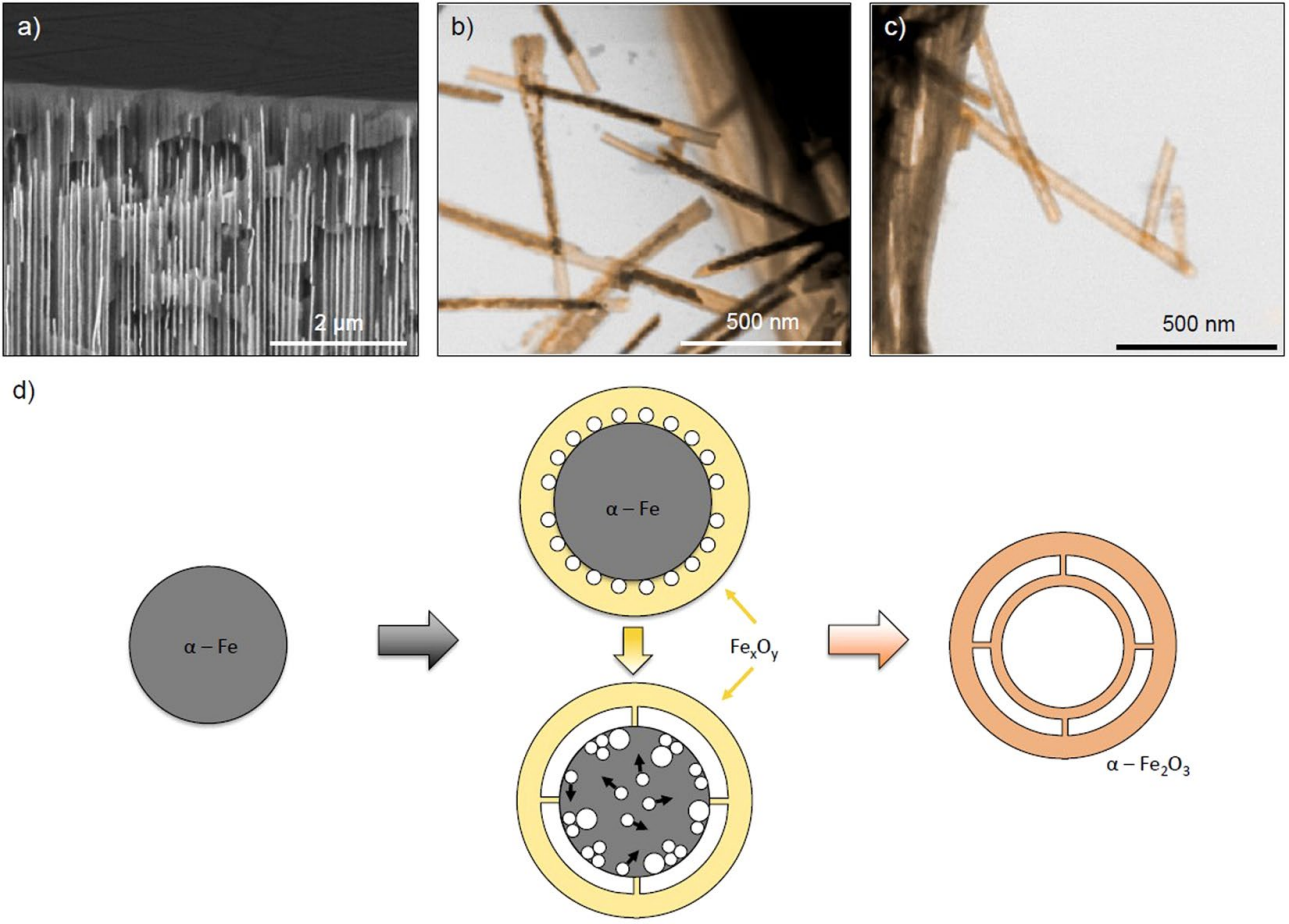
Morphology evolution of the nanostructures: SEM and STEM images of the products obtained (**a**) as prepared in template, (**b**) after chemical etching and (**c**) after annealing. STEM images were colored for better representation. (**d**) Schematic showing different stages of double-walled iron oxide formation: iron nanowire, iron oxide shell and selective coordination-assisted dissolution, void formation by the Kirkendall effect, complete oxidation of structure.

From STEM it is clear that a double-walled nanostructure is attainable by a simple chemical etching and annealing route. But the formation mechanism of these structures is not straightforward. The morphology of the samples at the various formation stages was studied and is presented in Fig. 5.

#### Formation of heterostructures by selective chemical etching

The as-deposited α-Fe sample had a typical structure of NWs fabricated through template-assisted electrodeposition, with no visible morphology defects (Fig. 5a). After several hours of selective chemical etching in a H_3_PO_4_ and H_2_Cr_2_O_7_ solution, the NWs were converted to a core-shell structure, with a very thin and smooth outer layer and a granular nucleus (Fig. 5b). In this step, two main processes occured: the formation of an iron oxide shell and the partial dissolution. When submerged for long times in an aqueous solution, the α-Fe nanowires slowly oxidized. A compact layer of iron oxide was formed on the surface of nanowires without visible gaps (Fig. 5b). The oxide shell was passive and stable in the aqueous solution^23^.

The shell diameter of ~46 nm was larger than the previous Fe NWs 35 nm diameter on the PAA template, imposed by the alumina template, which confirmed the oxidation of Fe accompanied with an expansion due to oxygen incorporation. During this intermediate stage, the nanowires break since they are no longer supported by the PAA. This yields aspect ratios ranging from ca. 22 to 109, that are the same as the final product, the double-walled nanotube. Optimization of the chemical etching step might decrease the nanowire breakage and further increase the final aspect ratio. XRD of the sample after alumina template removal (Fig. S2) indicated the presence of Fe and no trace of Fe oxide. The absence of Fe oxide phases was attributed to the amorphous nature of the outer wall that only crystallizes after annealing at high temperatures. After long exposures to etching solutions, the iron core was transformed due to the outward mass transport of iron to form the iron oxide shell combined with the dissolution of the iron shell. In this case, single-walled nanotubes can be achieved, as reported for hollow spherical nanoparticles^14,24–26^.

To achieve double-walled nanostructures, the α-Fe nucleus was preserved with a radius between 30–35 nm by decreasing the etching time to less than 20 h. It is observed that the remaining granular nucleus has several voids and a hollow tubular structure at the tips, left from the dissolved or detached nucleus (Fig. 5b). Based on this morphology and crystallography evolution between the previous steps, it is proposed a dissolution-based mechanism for the transformation of NW to core-shell heterostructure.

It has been demonstrated that iron oxide nanoparticles, in the presence of phosphate ions, promote a selective coordination-assisted dissolution^13^. Similarly, here Fe was first oxidized^27^ and then selectively dissolved. The presence of a smooth sheath and the voids along the structures at step 2 (Fig. 5b) supports the coordination-assisted dissolution mechanism preferentially in the grain boundaries of the iron core and in the tips of the tubes, where the oxidation of iron is a self-limiting process. Additionally, the flat outer oxide shell enables highly homogeneous and reproducible nanostructures, with many high-energy sites in the iron core that can be easily exposed to produce an internal nanotube.

#### Oxidation in high-temperature regime

The voids created during the etching process in the core of the heterostructures increase with the annealing ramp, as reported^28^. After 6 h of annealing at 600 °C the core structure is converted to an interior smooth oxide tube, inserted into the outer layer, created in the etching process (Fig. 5c). At this temperature the outer diameter increased once more, indicating a complete oxidation and crystallization of the structure. Annealing of Fe NWs in template was performed and no indication of tube formation was observed, as reported elsewhere^21^. On the other side, if the annealing is performed in dispersed nanoparticles or nanowires, the oxygen intake and Fe diffusion creates a hollow nanostructure^29,30^. Only if the annealing is performed on core-shell nanostructures a double-walled can be achieved. The inner wall formed during the annealing can be explained by the Kirkendall effect, where the rapid formation and growth of vacancies along the edge of the core compared to vacancy diffusion, leads to the conversion of the iron core into a secondary hematite tube^28,31–33^. STEM images show very tenuous connection between both tubes and it is here hypothesized that many small bridges are preserved during the vacancies formation and prevent both tubes to collapse. These connections are particular features of this method and its formation process is illustrated in Fig. 5d. With the increasing temperature, there is a continuous diffusion of the iron core and oxide shell through the interface between them, with consequent formation of excess vacancies at the center of the core and small Kirkendall voids at the iron/oxide interface^31^. Then, during the annealing time, the voids begin to collapse leading to the enlargement of the hollow core and the hollow space between the inner and the outer tubes where the skeletal bridges where preserved.

The proposed new methodology demonstrates that, if the annealing stage is performed after the NWs are immersed in a phosphate containing solution a double walled NT can be formed in a reproducible manner. Given that the template dictates the initial shape of the NW, a variety of aspect ratios should be obtained. Presently, various porous alumina templates are being tested with larger and smaller diameters. With recent advances in porous alumina template control, it should be even possible to use modulated template to a variety of length-to-diameter ratios for more complex applications^28,34^.

## Methods

### Sample preparation

Double-walled iron oxide NTs were prepared from arrays of metallic Fe NWs that were fabricated as described previously^22,35^. Briefly, a high purity thick Al foil was rinsed and electropolished to reduce surface roughness and create nanopatterns for posterior pore nucleation. The Al foils were subjected to a two-step anodization process to create a PAA template. The first one in 0.3 M oxalic acid, (COOH)_2_, at 40 V and 2 °C. Secondly, to obtain high aspect ratio NWs, a second anodization was carried out during 20 h (corresponding to 50 μm template thickness). Before electrodeposition, the anodization potential was lowered to 8 V to form dendritic channels^36^. Pulsed electrodeposition was employed to fill the template with Fe NWs in conditions previously optimized. A mixture of 153 g·L^−1^ Fe (II) sulfate heptahydrate, FeSO_4_·7H_2_O, 45 g·L^−1^ boric acid, H_3_BO_3_, and 1 g·L^−1^ ascorbic acid, C_6_H_8_O_6_, was used as a precursor in electrodeposition. A current density of 94 mA·cm^−2^ was applied. The obtained PAA templates were completely filled. To ensure that no Fe film was left on the surface of the template they were mechanically polished using alumina powder^22^.

Afterwards, the alumina template was dissolved in an aqueous solution of phosphoric acid, 0.4 M H_3_PO_4_, and dichromic acid, 0.2 M H_2_Cr_2_O_7_, at 60 °C (removal rate: 50 nm·min^−1^), resulting in core-shell nanostructures. These nanostructures were dispersed within an ultrasound bath and suspended in the previous solution during 1 h. After complete removal of the alumina template, the nanostructures were washed several times in ethanol. The dispersed nanostructures were then annealed in air at 600 °C for 6 h, at 2 °C/min heating and cooling ramps resulting in double-walled iron oxide NTs.

### Sample characterization

The morphology and surface topography of the samples were characterized using a high-resolution SEM in a FEI Quanta 400 FEG ESEM with an in-lens detector for secondary electrons. SEM imaging was carried out with an acceleration voltage of 15 kV and a working distance of about 10 mm. Cross-sectional images were acquired from freshly cleaved samples. STEM was performed in a FEI Titan 60–300 operated at 300 kV and equipped with an aberration corrector for the probe. STEM spectrum imaging combining HAADF imaging and EELS was used for the local chemical analysis of the NTs. Chemical line profiles and maps of the NTs were obtained from EELS spectrum images by integrating the O-K and Fe L_2,3_ edges intensity after background extrapolation and subtraction.

The crystalline structure of the samples was analyzed by XRD in a Rigaku SmartLab diffractometer in the θ − 2θ parallel beam geometry using the Cu − Kα line with wavelength λ = 1.540593 Å. The spectra were acquired by moving the detector between 2θ = [20°, 80°] at a scan rate of 0.5 deg·min^−1^ with a step width of 0.04°. The spectra peaks were fitted using a Lorentzian function to determine the average grain size according to the Scherrer equation, as described elsewhere^35^. Reflection patterns were matched to reference values – Table S1. To measure the crystalline structure of Fe NWs in the template, the Al substrate was removed to eliminate its contribution. For that, the samples were submerged in 0.5 M CuCl_2_ and 2.8 M HCl solution.

Raman spectra were determined on a LabRAM by Jobin-Yvon micro-sampling with the excitation wavelength of 633 nm of a He-Ne laser at 100x amplification. To avoid sample damaging the laser power was 10 mW. Gratings of 1800 gr/mm were used. The measurements were from 200 to 1200 cm^−1^ using a 100 μm slit. Each measurement was done over 30 s at two different places in each sample to assure a correct reproducibility of the spectrum. The samples were put in a low-susceptibility plastic sample holder for measurements. X-ray absorption spectroscopy measurements at the Fe K-edge in in transmission were developed at the XAFS beamline of the Elettra synchrotron with an energy range of 6812–7969 eV. An Fe metal foil spectrum was also recorded for energy calibration. For each sample, three spectra were acquired for statistics with an integration time of 5.00 s. The experimental XANES spectrum of the samples was normalized at high energy after background subtraction to eliminate thickness dependence. To quantify the composition of each sample, the spectra were fitted with reference Fe/Fe oxide absorption curves using a specifically made script.

## Conclusions

Using a simple and low-cost fabrication method, the synthesis of double-walled hematite nanotubes was achieved using porous anodic alumina templates to control the overall morphology of the nanotubes. The features of these structures were assessed by STEM revealing diameters smaller than 50 nm and wall thicknesses less than 10 nm, in addition to a subtle network of connection bridges between inner and outer nanotube walls. The crystallography and composition were evaluated precisely revealing a single iron oxide phase – hematite. Finally, the growth mechanism of these double-walled nanotubes was revealed. A synergetic combination of a selective chemical etching and annealing on metallic nanowires allowed the fabrication of double-walled oxide nanotubes with controlled morphology.

## Supplementary information

Supplementary Information
