# Supplementary material for: Double-walled iron oxide nanotubes via selective chemical etching and Kirkendall process
Source: Sci Rep. 2019 Aug 19;9:11994. doi: 10.1038/s41598-019-47704-5 (PMC6700129; doi:10.1038/s41598-019-47704-5)
Supplement: Supplementary file 1 — Supplementary Information [file 41598_2019_47704_MOESM1_ESM.docx]

*Supporting Information*

Double-walled iron oxide nanotubes via selective chemical etching and Kirkendall process

João Azevedo^1^ Maria P. Fernandez-García^2^, César Magén^3^, Adélio Mendes^1^, João P. Araújo^2^, Célia T. Sousa*^,2^

1. LEPABE - Faculdade de Engenharia, Universidade do Porto, Rua Dr. Roberto Frias, 4200-465 Porto, Portugal

2. IFIMUP and Departamento de Física e Astronomia da Faculdade de Ciências da Universidade do Porto, Rua do Campo Alegre 687, 4169-007 Porto, Portugal

3. Instituto de Ciencia de Materiales de Aragón (ICMA), Universidad de Zaragoza-CSIC, 50009 Zaragoza, Spain and Laboratorio de Microscopías Avanzadas (LMA) - Instituto de Nanociencia de Aragón (INA), Universidad de Zaragoza, 50018 Zaragoza, Spain and Departamento de Física de la Materia Condensada, Universidad de Zaragoza, 50009 Zaragoza, Spain.

* celiasousa@fc.up.pt

**
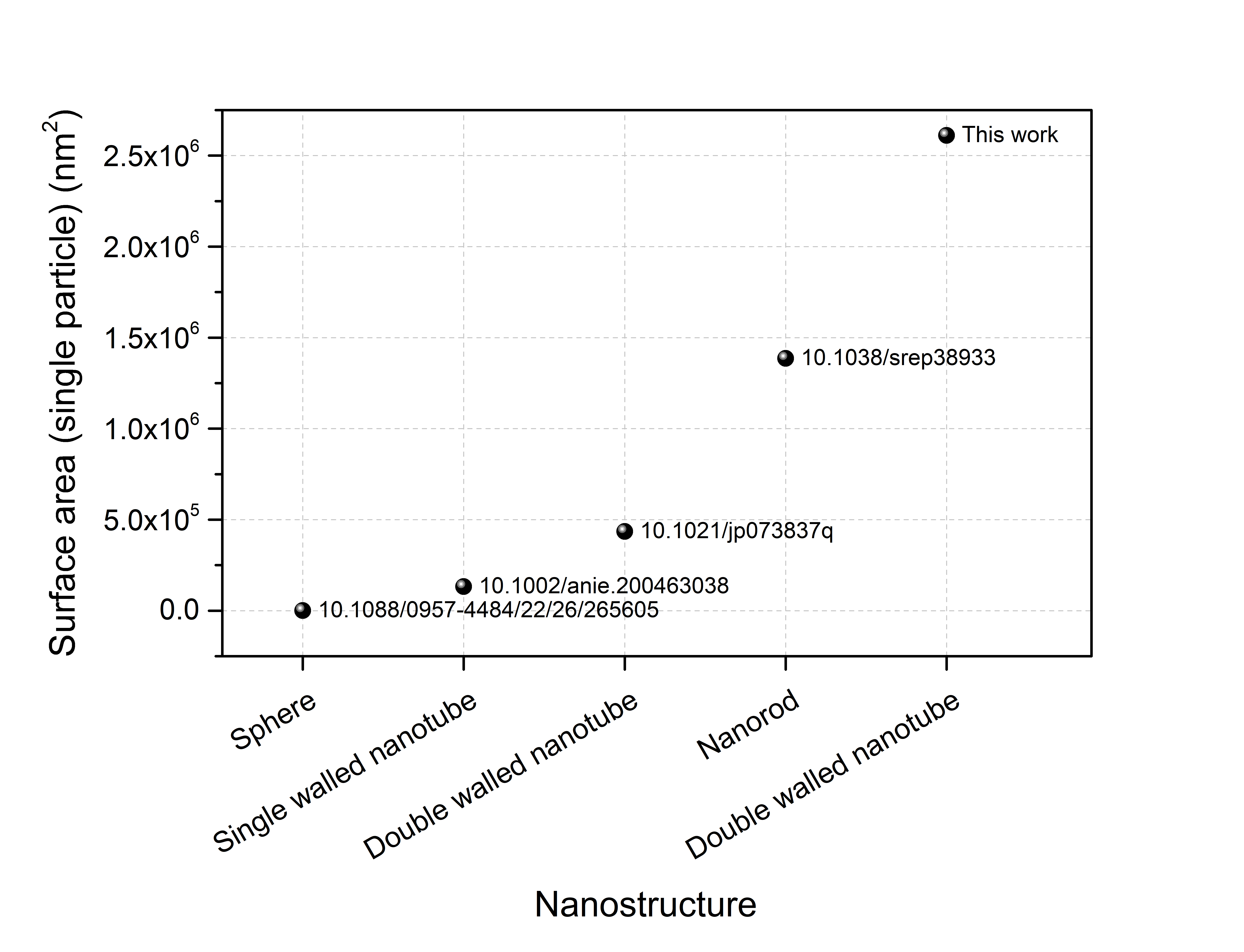
**

**Figure S1.** Approximate geometric surface area for a single dispersed nanoparticle with different geometries. For each geometry, a single particle was considered, since in some references only one high resolution TEM image was shown, and approximated to a perfect geometric shape (for example, a nanotube was approximated to a perfect tube with no irregularities). Next to each data point, the DOI of the corresponding reference is mentioned.

**Figure S2.** X-ray diffraction (XRD) patterns with corresponding layer Bragg reflections of nanoparticles in template and after alumina template removal. Peak values were compared to reference – Table S1.

**Table S1.** Structural parameters calculated from the XRD patterns and reference values (PDF card nº 2101167) for the diffracting planes.

| **(*h k l*) crystal system** | **2*θ*** | |
| --- | --- | --- |
|  | **XRD** | **Reference values** |
| 0 1 2 | 24.186 | 24.15 |
| 1 0 4 | 33.119 | 33.16 |
| 1 1 0 | 35.622 | 35.63 |
| 1 1 3 | 40.944 | 40.86 |
| 2 0 2 | 43.393 | 43.51 |
| 0 2 4 | 49.562 | 49.46 |
| 1 1 6 | 54.268 | 54.07 |
| 1 2 2 | 57.743 | 57.61 |
| 2 1 4 | 62.640 | 62.44 |
| 3 0 0 | 64.186 | 64.00 |
| 1 0 10 | 72.175 | 71.96 |
| 2 2 0 | 75.732 | 75.45 |
